# Diplacone Isolated from *Paulownia tomentosa* Mature Fruit Induces Ferroptosis-Mediated Cell Death through Mitochondrial Ca^2+^ Influx and Mitochondrial Permeability Transition

**DOI:** 10.3390/ijms24087057

**Published:** 2023-04-11

**Authors:** Myung-Ji Kang, Hyung Won Ryu, Eun Sol Oh, Yu Na Song, Yang Hoon Huh, Ji-Yoon Park, Seon Min Oh, Su-Yeon Lee, Yhun Jung Park, Doo-Young Kim, Hyunju Ro, Sung-Tae Hong, Su Ui Lee, Dong-Oh Moon, Mun-Ock Kim

**Affiliations:** 1 Natural Product Research Center, Korea Research Institute of Bioscience and Biotechnology (KRIBB), Cheongju 28116, Republic of Korea; mjkang@kribb.re.kr (M.-J.K.); ryuhw@kribb.re.kr (H.W.R.); zkx2@kribb.re.kr (E.S.O.); alsrud5354@kribb.re.kr (Y.N.S.); parkgyun98@kribb.re.kr (J.-Y.P.); seonmin88@kribb.re.kr (S.M.O.); lsy802@kribb.re.kr (S.-Y.L.); akdge@kribb.re.kr (Y.J.P.); rose73@kribb.re.kr (D.-Y.K.); iamsuui@kribb.re.kr (S.U.L.); 2 Department of Biological Sciences, College of Bioscience and Biotechnology, Chungnam National University, Daejeon 34134, Republic of Korea; rohyunju@cnu.ac.kr; 3 Korea Basic Science Institute, Cheongju 28119, Republic of Korea; hyh1127@kbsi.re.kr; 4 Departments of Anatomy & Cell Biology, Department of Medical Science, College of Medicine, Chungnam National University, Daejeon 35015, Republic of Korea; mogwai@cnu.ac.kr; 5 Department of Biology Education, Daegu University, 201 Daegudae-ro, Gyeongsan-si 38453, Republic of Korea

**Keywords:** diplacone (DP), mitochondria permeability transition (MPT), ferroptosis, vacuolation

## Abstract

The recently defined type of cell death ferroptosis has garnered significant attention as a potential new approach to cancer treatment owing to its more immunogenic nature when compared with apoptosis. Ferroptosis is characterized by the depletion of glutathione (GSH)/glutathione peroxidase-4 (GPx4) and iron-dependent lipid peroxidation. Diplacone (DP), a geranylated flavonoid compound found in *Paulownia tomentosa* fruit, has been identified to have anti-inflammatory and anti-radical activity. In this study, the potential anticancer activity of DP was explored against A549 human lung cancer cells. It was found that DP induced a form of cytotoxicity distinct from apoptosis, which was accompanied by extensive mitochondrial-derived cytoplasmic vacuoles. DP was also shown to increase mitochondrial Ca^2+^ influx, reactive oxygen species (ROS) production, and mitochondrial permeability transition (MPT) pore-opening. These changes led to decreases in mitochondrial membrane potential and DP-induced cell death. DP also induced lipid peroxidation and ATF3 expression, which are hallmarks of ferroptosis. The ferroptosis inhibitors ferrostatin-1 and liproxstatin-1 were effective in counteracting the DP-mediated ferroptosis-related features. Our results could contribute to the use of DP as a ferroptosis-inducing agent, enabling studies focusing on the relationship between ferroptosis and the immunogenic cell death of cancer cells.

## 1. Introduction

Lung cancer is a noteworthy cause of cancer-related fatalities across the globe, with non-small-cell lung cancer accounting for 84% of cases and small-cell lung cancer comprising 13% of cases, distinguished by their size and type [1]. The pathological features and treatment protocols of these two types of cancer vary significantly. In particular, advanced-stage non-small-cell lung cancer is usually treated by chemotherapy or immunotherapy [2].

Cell death is categorized as regulated cell death (RCD) and accidental cell death (ACD), with RCD being able to regulate cell death through specific regulatory molecules. In recent years, various RCD pathways have been elucidated, including programmed cell death (PCD), intrinsic apoptosis, necroptosis, pyroptosis, NETosis, parthanatos, mitochondrial permeability transition (MPT)-regulated necrosis, and ferroptosis, through extensive studies spanning the last 10–20 years [3,4]. Ferroptosis is a type of cell death that depends on ferrous (Fe^2+^) ions, and one of its key features is the buildup of excessive lipid peroxidation in the cell membrane. The process of lipid peroxidation can be initiated by either an enzymatic reaction catalyzed by lipoxygenase (LOX) or a non-enzymatic reaction. Once the lipid peroxide is produced, it can react with Fe^2+^, oxygen, or surrounding unsaturated fatty acids, leading to the continuous generation of radicals that spread to surrounding areas and trigger cell death [5]. Although unsaturated fatty acids in the cell membrane are constantly oxidized, even under normal conditions, glutathione peroxidase 4 (GPX4) works to prevent the accumulation of lipid peroxide and thus protect cells from ferroptosis. GPX4 is an enzyme that catalyzes the oxidation of 2GSH to produce GSSH while also reducing lipid peroxide to produce lipid alcohol. There are two major groups of drugs that induce ferroptosis. Class I FINs (Ferroptosis Inducing agents) include Erastin, Sulfasalazine, and Sorafenib, which block system xc^−^. System xc^−^ is a cystine–glutamate antiporter that imports the amino acid cystine into cells. Treatment with class I FINs induces ferroptosis by causing cysteine and glutathione (GSH) deficiency in cells. Class II FINs, such as RSL3, ML210, and ML162, inhibit GPX4 and also induce ferroptosis through the accumulation of lipid peroxidation [6,7]. Ferroptosis is a newly recognized form of immunogenic cell death that triggers secondary apoptosis by releasing damage-associated molecular patterns (DAMPs) from the cell [8,9]. When cells undergo ferroptosis, they secrete HMGB1, a type of DAMP that activates a pattern recognition receptor (PRR)/NF-κB pathway, leading to an inflammatory response in peripheral macrophages [10,11].

The formation of a permeability transition pore complex (PTPC) between the inner and outer mitochondrial membranes is the cause of mitochondrial permeability transition (MPT)-driven necrosis due to changes in the intracellular microenvironment, such as oxidative stress or excess Ca^2+^ [12,13]. The composition, regulation, and precise mechanism of PTPC are still unclear and controversial. The only known protein required to induce MPT-driven necrosis in vivo is peptidylprolyl isomerase F (PPIF; cyclophilin D) [14]. Cyclosporin A, an inhibitor of cyclophilin D, can inhibit the development of MPT-driven necrosis in diseased animals with cardiac and cerebral ischemia [15]. Based on these findings, we hypothesize that ferroptosis induces MPT-driven necrosis, which results in an increase in HMGB1 secretion in lung epithelial A549 cells, ultimately leading to greater cytotoxicity.

In the past, the fruit of *Paulownia tomentosa* (Thunb.) Steud. (Paulowniaceae) has been used in Asia as a traditional remedy for various diseases, such as tonsillitis, bronchitis, asthmatic attacks, enteritis, and dysentery [16]. In a previous study by our colleagues, diplacone (DP) was isolated from methanol extracts of dried *P. tomentosa* fruits, along with 18 other dihydroflavonols and flavanone derivatives [16]. DP has been reported to have anti-inflammatory [17], antioxidant [18], anticancer [19], and antibacterial properties [20,21]. DP was found to have an antiproliferative effect on human ovarian cancer cell line A2780 [22], but the cellular mechanism of the action of DP has not yet been fully elucidated. In this study, we observed that DP treatment of A549 human non-small lung carcinoma cells led to the formation of extensive cytoplasmic vacuoles and decreased cell growth, prompting us to investigate the underlying cellular mechanisms by which DP induces cancer cell death and causes vacuole formation in A549 cells.

## 2. Results

### 2.1. DP Induces Cell Death with Extensive Cytoplasmic Vacuoles in A549 Cells

**Figure 1:**
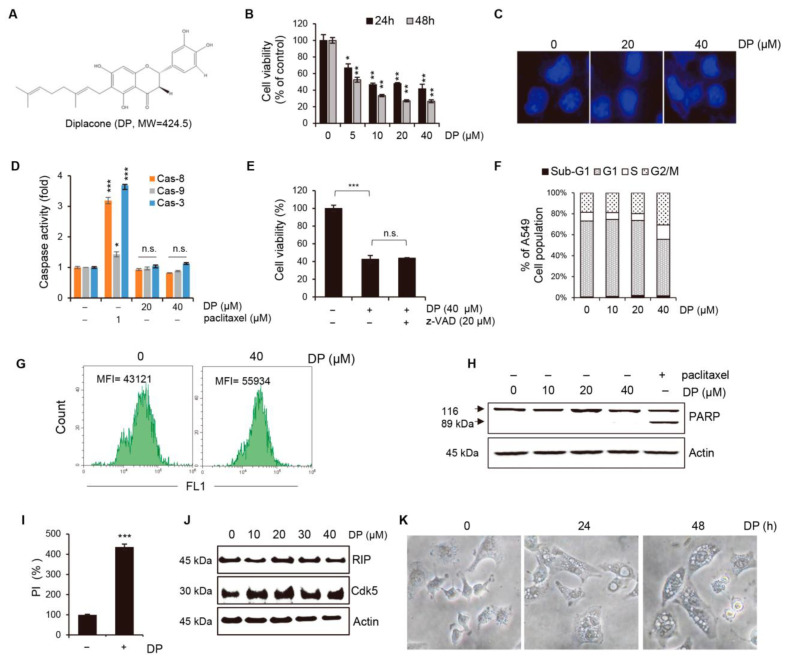
Apoptosis is not critically involved in DP-induced cancer death. (**A**) This shows the molecular structure of DP. (**B**) A549 non-small lung cancer cells were treated with the indicated concentrations of DP for 24 or 48 h and cell viability was measured using the MTT assay. (**C**) Nuclear fragmentation was visualized under a fluorescence microscope (magnification, 100×; scale bar, 0.1 mm). (**D**) The in vitro activities of caspase-8, caspase-9, and caspase-3 were measured in A549 cells lysed after 24 h of treatment with the indicated concentrations of DP. Paclitaxel (1 μM) was used as a positive control. (**E**) To inhibit pan-caspase, A549 cells were pretreated with 20 µM of z-VAD-fmk for 1 h, and after 24 h of DP treatment at the indicated concentration, cell viability was measured by the MTT assay. (**F**) Flow cytometry was used to analyze the cell cycle after DP treatment at various concentrations for 24 h. (**G**) The TUNEL assay was used to evaluate the apoptosis levels in A549 cells following a 24 h treatment with 40 μM DP. (**H**) A549 cells were treated with indicated concentration of DP for 24 h, and Western blotting was performed to assess PARP cleavage. Actin was used as a loading control. (**I**) PI-stained cells were detected using flow cytometry after 24 h of 40 µM DP treatment. (**J**) A549 cells were treated with the indicated concentration of DP for the indicated time, and Western blotting was performed to assess RIP and Cdk5 expression. Actin was used as a loading control. (**K**) Morphological changes of A549 cells were observed under a microscope after 40 µM DP treatment (magnification, ×200). Statistical differences were analyzed by two-way ANOVA with Tukey’s multiple comparisons test and presented as *p* < 0.05 (*), *p* < 0.01 (**), and *p* < 0.001 (***) when compared with the DMSO control. Different lowercase letters above the bar graph indicate significant statistical differences (*p* < 0.05). n.s.: not significant.

We separated and examined 19 dihydroflavonol and flavanone compounds from ripe *P. tomentosa* fruits (Supplementary Figure S1). Among these, a cytotoxic substance, designated as DP, was isolated and purified. DP’s structure is depicted in Figure 1A. To evaluate the effect of DP on human lung cancer A549 cell viability, we treated cells with different concentrations of DP for 24 or 48 h and performed the MTT assay. The results showed that DP treatment significantly inhibited cell growth in a dose-dependent manner in A549 cells (Figure 1B). Our results show that the IC50 values of DP against A549 cells were 10.6 µM and 7.9 µM at 24 and 48 h, respectively. We then investigated several apoptotic features to determine whether the decrease in A549 cell growth was due to apoptosis. We performed Hoechst 33,342 staining to label nuclear DNA in living cells after DP treatment and observed no condensed or segmented nuclei (Figure 1C). Caspases represent an endoprotease family playing an essential role in apoptosis. Caspases are classified as initiator caspases (caspase-8 and caspase-9) or executioner caspases (caspase-3, caspase-6, and caspase-7), depending on their stage of action [23]. We investigated the proteolytic activity of caspase-8, caspase-9, and caspase-3, and found that there was a slight change in their activity in the DP 40 μM-treated group, although the change was not significant (Figure 1D). The administration of z-VAD-fmk, a pan-caspase inhibitor, failed to reverse the cell death induced by 40 µM DP treatment (Figure 1E). In addition, DP did not induce any concentration-dependent arrest in A549 cells at certain cell cycle phases or in an apoptotic sub-G1 population (Figure 1F). Furthermore, to confirm whether DP induced apoptosis, a TUNEL assay was conducted. However, there was no observed increase in FITC fluorescence upon the addition of UTP (Figure 1G). Next, to clarify whether DP induced apoptosis, PARP was confirmed by Western blotting. PARP (Poly(ADP-ribose) polymerase) has been shown to play a role in apoptosis, a programmed form of cell death. During apoptosis, PARP can be cleaved by caspase-3 and other caspases, resulting in the inactivation of PARP and the release of its fragments. These fragments, known as PARP cleavage products, have been proposed to regulate various aspects of the apoptotic process. As shown in Figure 1H, treatment with DP did not induce the cleavage of PARP. Therefore, we used the PI uptake assay to determine whether necrotic cell death was induced. The results showed that PI uptake was increased in 40 µM DP-treated cells, which was thought to be due to plasma membrane rupture caused by necrotic cell death (Figure 1I). To confirm whether PI uptake by DP was due to necrosis, RIP and Cdk5, which are necrosis-related proteins, were confirmed by Western analysis. RIP, which stands for receptor-interacting serine/threonine-protein kinase, is a key player in necrosis. The kinase activity of RIP1 can trigger the formation of a protein complex known as the necrosome, which can activate downstream proteins, such as MLKL (mixed-lineage kinase domain-like protein). Ultimately, this leads to the breakdown of the plasma membrane and the release of intracellular contents, resulting in cell death. In addition, recent studies have suggested that Cdk5 may also contribute to necrosis by regulating mitochondrial function. As shown in Figure 1J, DP treatment did not affect the expression of necrosis-related proteins. Interestingly, DP-treated A549 cells exhibited extensive cytoplasmic vacuolation that could be observed under a microscope (Figure 1K).

### 2.2. The Origin of Vacuoles Is Mitochondria

**Figure 2:**
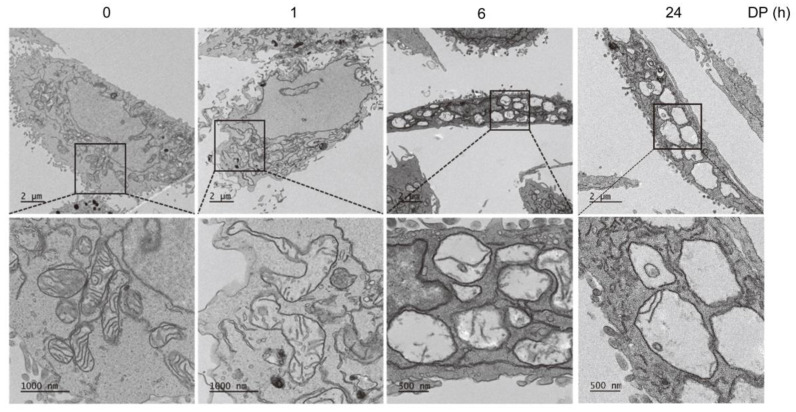
DP-induced mitochondrial expansion. After treatment with 40 µM DP for 1, 6, and 24 h, the A549 cells were observed using transmission electron microscopy.

We investigated the A549 cells with higher-level magnification using transmission electron microscopy (TEM) and found that the cytoplasmic vacuoles induced by DP originated from the mitochondria (Figure 2). A549 cells treated with DP did not display apoptotic nuclear fragmentation, and instead showed the presence of multiple rounded mitochondria with damaged internal cristae, as observed morphologically. The results showed that DP treatment led to a time-dependent increase in mitochondrial expansion, with a distinct rise observed between 1 and 24 h after treatment. Furthermore, it was observed that the mitochondria increased in size and the frequency of empty vacuoles also significantly increased. In the case of macroautophagosomes generated by autophagy, vacuoles can be observed with the naked eye, so we checked whether autophagy was induced by DP treatment. Autophagy inhibitors, including Wortmannin, Chloroquine, and Bafilomycin A1, did not prevent DP-induced cell death (Supplementary Figure S2A). ATG5 knockout cells did not affect DP-induced cell viability (Supplementary Figure S2B). Taken together, these findings indicate that vacuole formation driven by DP is not a result of autophagy activation.

### 2.3. DP Promotes Cell Death through Increasing Cytosolic Ca^2+^ Concentration

**Figure 3:**
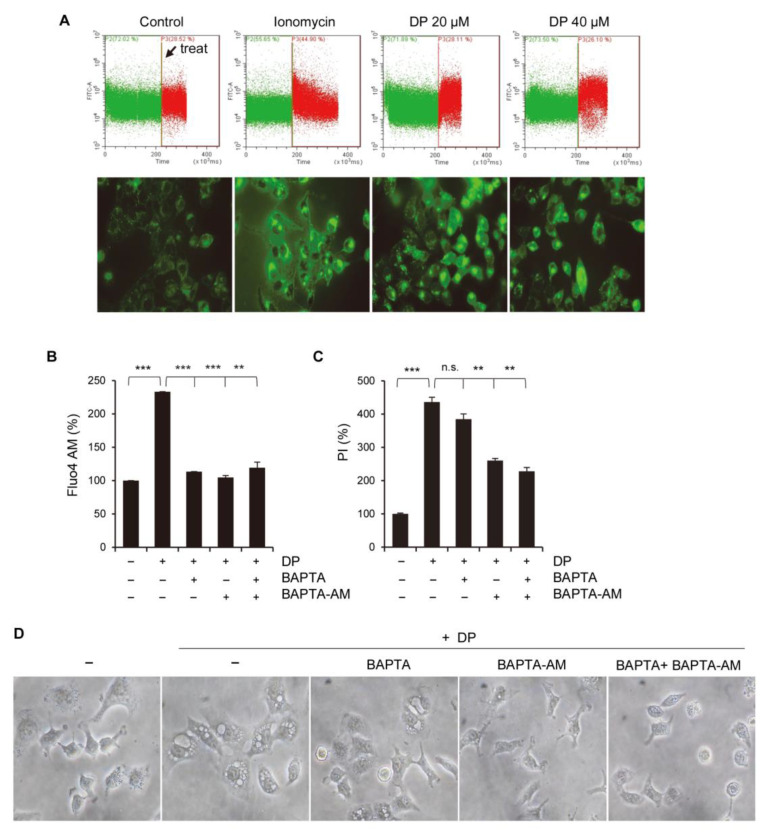
DP increases cytosolic Ca^2+^. (**A**) A549 cells were preloaded with 2 μg/mL Fluo-4 AM and incubated with 20 and 40 μM DP for 1 min to examine the changes in intracellular Ca^2+^ using flow cytometry (upper panel) and a fluorescence microscope (lower panel). Ionomycin (1 μg/mL) was used as a positive control. (**B**) A549 cells were pretreated with 10 µM BAPTA, 10 µM BAPTA-AM, or 10 µM BAPTA plus 10 µM BPATA-AM, followed by treatment with 40 µM DP for 1 min. The cells were treated with 0.5 µg/mL Fluo-4 AM and analyzed using flow cytometry. (**C**) A549 cells were pretreated with Ca^2+^ chelators, treated with 40 µM DP for 24 h, and the PI intensity was measured by flow cytometry. (**D**) A549 cells were pretreated with Ca^2+^ chelators and further treated with 40 µM DP for 6 h. Cell morphological changes were observed under a microscope (magnification, ×200). All data are expressed as the mean ± SD obtained from at least three independent experiments. Statistical differences were analyzed by two-way ANOVA with Tukey’s multiple comparisons test and presented as *p* < 0.01 (**) and *p* < 0.001 (***) compared with the control. n.s.: not significant.

The intracellular Ca^2+^ concentration was measured by flow cytometry using fluorescent Fluo-4 AM, a cell-permeable Ca^2+^ indicator dye. An increase in cytosolic calcium concentration was observed in the cells treated with DP, as well as ionomycin used as a positive control (Figure 3A). Next, we used Ca^2+^ chelators to determine whether Ca^2+^ influx was associated with DP-induced cell death. BAPTA (an extracellular Ca^2+^ scavenger) or BAPTA-AM (a cell-permeable acetoxymethyl ester Ca^2+^ chelator) significantly attenuated the increase in intracellular Ca^2+^ (Figure 3B). Additionally, BAPTA and BAPTA-AM pretreatments slightly, but significantly, reversed the 40 μM DP treatment-induced PI staining (Figure 3C). After the A549 cells were treated with DP, morphological changes were observed under a microscope. BAPTA, BAPTA-AM, and BAPTA plus BAPTA-AM pretreatments inhibited DP-induced cellular vacuolation (Figure 3D). These results indicate that DP-induced A549 cell death and the formation of cytoplasmic vacuolation are closely associated with the increased intracellular Ca^2+^ level.

### 2.4. DP Increases Mitochondrial Ca^2+^ Overload That Critically Involved in DP-Induced Cell Death

**Figure 4:**
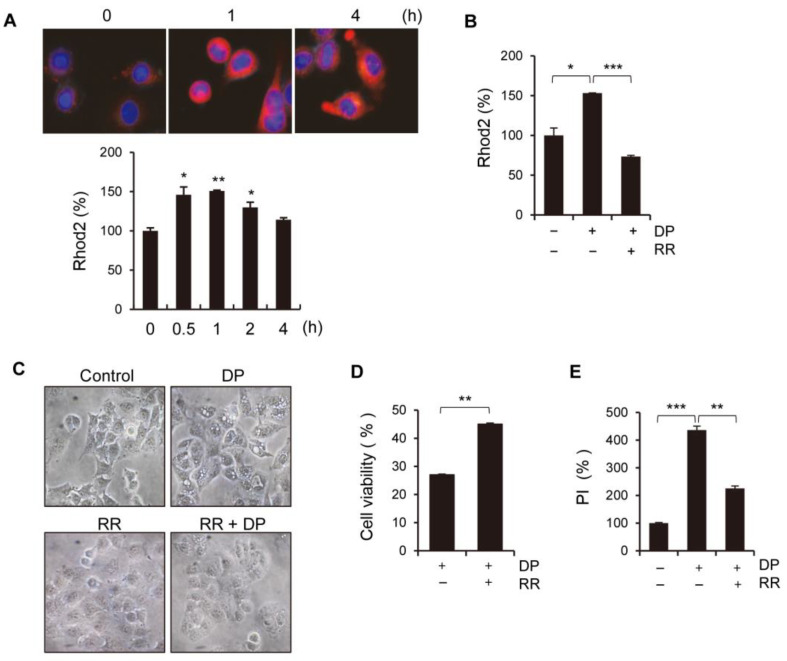
DP induces Ca^2+^ uptake in mitochondria. (**A**) A549 cells were treated with 40 µM DP for the indicated periods and stained with 1 µg/mL Rhod-2 for fluorescence microscope (upper panel) and flow cytometry (lower panel) analysis. Nuclei were stained with Hoechst 33242 (blue) and mitochondrial Ca^2+^ were stained with Rhod-2 (red). (**B**) The A549 cells were pretreated with 5 µM ruthenium red (RR) and then treated with 40 µM DP. (**C**) The cells were pretreated with RR and further treated with DP, and cell morphology was observed under a microscope (magnification, ×200). (**D**) The cells were pretreated with RR and then treated with DP for 24 h, and cell viability was assessed using the MTT assay. (**E**) The cells were pretreated with RR and then treated with DP for 24 h, and PI-positive cells were analyzed via flow cytometry. All data are expressed as the mean ± SD obtained from at least three independent experiments. Statistical differences were analyzed by two-way ANOVA with Tukey’s multiple comparisons test and presented as *p* < 0.05 (*), *p* < 0.01 (**), and *p* < 0.001 (***) when compared with the control.

More specifically, we examined whether DP could affect Ca^2+^ influx into mitochondria. Using Rhod-2, an indicator dye for mitochondrial Ca^2+^ presence, we observed that the mitochondrial Ca^2+^ levels, confirmed by a fluorescence microscope and flow cytometry, were highest 1 h after the DP treatment and gradually decreased over time (Figure 4A). Cytoplasmic Ca^2+^ approaches the outer mitochondrial membrane via voltage-dependent anion-selective channels (VDAC) and is transported through the inner mitochondrial membrane via mitochondrial calcium uniporters (MCUs), rapid mitochondrial calcium uptake, or mitochondrial ryanodine receptors [24]. We investigated the alterations promoted by DP in A549 cells, such as enhanced mitochondrial Ca^2+^ absorption and vacuolization, which were counteracted by pre-treatment with ruthenium red (RR), an inhibitor of uniporter-mediated mitochondrial Ca^2+^ uptake and a non-selective MCU blocker. As a result of the Rhod-2 staining, RR pretreatment significantly blocked DP-facilitated Ca^2+^ influx into mitochondria (Figure 4B). Surprisingly, RR inhibited the DP-induced extensive vacuole formation in the cytoplasm, suggesting that Ca^2+^ influx into mitochondria is closely related to vacuole formation (Figure 4C). Additionally, we observed that RR pretreatment significantly reversed DP-induced cell death and PI staining (Figure 4D,E). In summary, these findings suggest that Ca^2+^ influx into mitochondria plays a critical role in DP-induced cell death, as well as extensive cytoplasmic vacuole formation, in A549 cells.

### 2.5. Increased Mitochondrial Ca^2+^ Influx by DP Triggers Increased Mitochondrial ROS Levels

**Figure 5:**
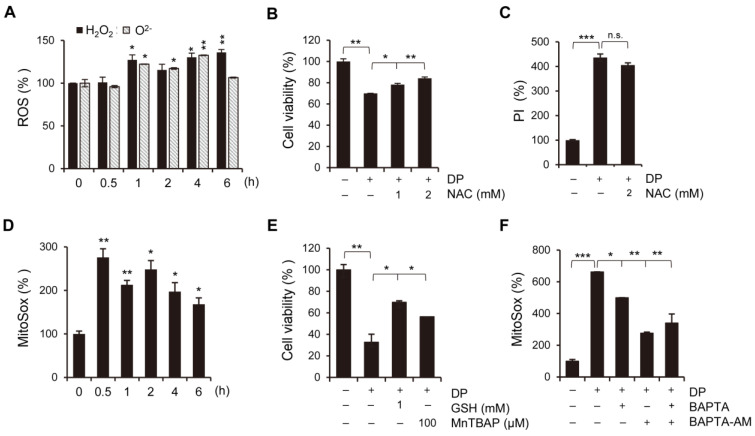
Mitochondrial Ca^2+^ overload acts upstream of ROS production. (**A**) A549 cells were treated with 40 µM DP for the indicated time. The cells were exposed to the hydrogen peroxide-sensitive dye H_2_DCF-DA and the superoxide-sensitive dye HE at 37 °C for 20 min, and ROS production was then analyzed using flow cytometry. (**B**,**C**) A549 cells were pretreated with NAC and then further treated with 40 µM DP. Cell growth was analyzed by the MTT assay (**B**), and the degree of PI staining was analyzed by flow cytometry (**C**). (**D**) A549 cells were treated with 40 µM DP for the indicated time. The treated cells were incubated with MitoSOX for 20 min at 37 °C in the dark and analyzed via flow cytometry. (**E**) A549 cells pretreated with GSH and MnTBAP, and further treated with 40 µM DP for 24 h. Viability was assessed using the MTT assay. (**F**) A549 cells pretreated with Ca^2+^ chelators and then incubated with MitoSOX for 20 min at 37 °C in the dark and analyzed via flow cytometry. All data are expressed as the mean ± SD obtained from at least three independent experiments. Statistical differences were analyzed by two-way ANOVA with Tukey’s multiple comparisons test and presented as *p* < 0.05 (*), *p* < 0.01 (**), and *p* < 0.001 (***) compared with the control. n.s.: not significant.

Generally, a high Ca^2+^ load is closely related to excessive ROS production in the mitochondria [25]. To observe the changes in the ROS levels, we performed flow cytometry analysis using H_2_DCF-DA and HE, fluorescent probes for intracellular ROS detection. We confirmed that DP treatment gradually increased the concentration of cytoplasmic ROS, including superoxide anions and hydrogen peroxide, in A549 cells (Figure 5A). It is chemically assumed that ROS may originate from the mitochondria, considering the stepwise O_2_ reduction process after encountering a free radical (O_2_ → O_2_^−^ → H_2_O_2_ → H_2_O). NAC (an antioxidant) pretreatment inhibited DP-induced cell death, but not PI uptake (Figure 5B,C). We predicted that irreversible cell death was induced by the influence of Ca^2+^ on an upstream mechanism that was expected to attenuate the response of ROS inhibitors. Next, we measured the mitochondrial ROS levels using MitoSOX, a mitochondrial superoxide anion indicator. As shown in Figure 5D, the mitochondrial O_2_^−^ levels increased dramatically, reaching a peak 0.5 h after DP treatment, and then gradually decreased. We also observed that pretreating the cells with GSH and MnTBAP, a superoxide anion scavenger, restored DP-induced cell death (Figure 5E). Interestingly, BAPTA and BAPTA-AM treatment significantly decreased the DP-induced mitochondrial ROS increase (Figure 5F). These results suggest that DP exposure-induced Ca^2+^-dependent ROS generation in the mitochondria implies irreversible mitochondrial dysfunction.

### 2.6. DP Induces Loss of Mitochondrial Membrane Potential

**Figure 6:**
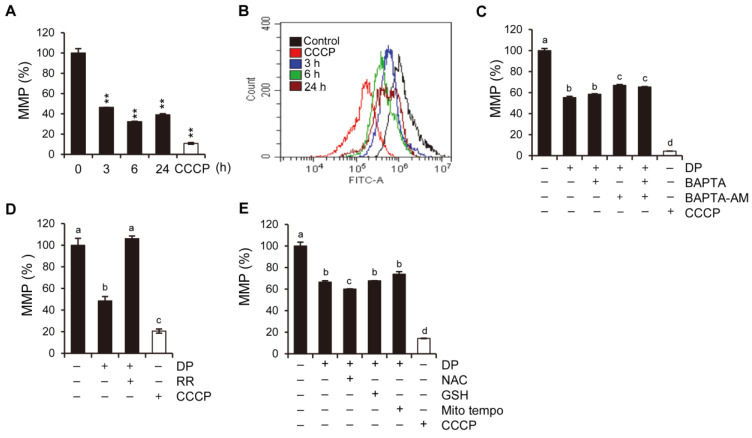
DP depletes mitochondrial membrane potential in A549 cells. (**A**) Carbonyl cyanide 3-chlorophenylhydrazone (CCCP; 50 µM) was used as a positive control. (**B**) A549 cells were treated with 40 µM DP for the indicated time, then stained with 40 nM DiOC6(3) for 30 min at 37 °C and analyzed via flow cytometry. (**C**–**E**) A549 cells were cotreated or pretreated with the indicated Ca^2+^ chelator, RR, and NAC and GSH for 1 h and further treated with 40 µM DP for 6 h. MMP was assessed using DiOC6(3). All data are expressed as the mean ± SD obtained from at least three independent experiments. Statistical differences were analyzed by two-way ANOVA with Tukey’s multiple comparisons test and presented as *p* < 0.01 (**) compared with the control. Different lowercase letters above the bar graph indicate significant statistical differences (*p* < 0.05).

To determine whether DP affects mitochondrial function, we investigated the changes in the mitochondrial membrane potential (MMP) after DP treatment using 3,3’-dihexyloxacarbocyanine iodide (DiOC6(3)). DiOC6(3) is a cell-permeable and lipophilic dye that accumulates in the mitochondria due to their large negative membrane potential and can be used to monitor the MMP. The MMP in A549 cells was significantly reduced and essentially lost 3 h after CCCP treatment, lasting up to 24 h, with CCCP used as a positive control (Figure 6A). As shown in Figure 6B, it was confirmed that MMP was reduced in A549 cells treated with 20 µM DP for 3, 6, and 24 h. Pretreatment with BAPTA and BAPTA-AM partially reversed the DP-induced MMP loss (Figure 6C). Surprisingly, pretreatment with the mitochondrial Ca^2+^ uptake blocker RR resulted in almost complete blocking of DP-induced MMP reduction (Figure 6D). However, ROS scavenger treatment did not reverse the DP-mediated MMP reduction (Figure 6E), possibly due to the excessive DP-induced Ca^2+^ overload strongly excreted upstream. These results indicate that DP-induced mitochondrial Ca^2+^ overload triggers MMP dissipation.

### 2.7. DP-Induced Cell Death Is Associated with MPT Pore-Opening

**Figure 7:**
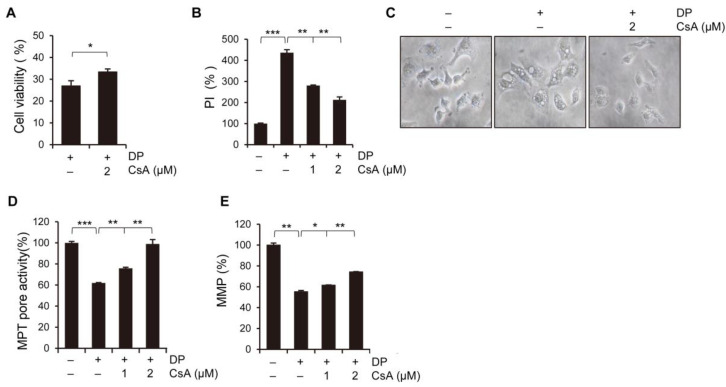
DP induces necrotic cell death via MPT-mediated mitochondrial Ca^2+^ influx in A549 cells. The cells were treated with 40 μM DP for 24 h with 1 h pretreatment of Cyclosporin A (CsA). (**A**) Cell growth was measured by the MTT assay. (**B**) PI-positive cells were analyzed via flow cytometry. (**C**) Cell shape was observed through an optical microscope (magnification, ×200). (**D**) MPT pores were measured using an MPT pore assay kit. (**E**) MMP was measured using DiOC6(3). All data are expressed as the mean ± SD obtained from at least three independent experiments. Statistical differences were analyzed by two-way ANOVA with Tukey’s multiple comparisons test and presented as *p* < 0.05 (*), *p* < 0.01 (**), and *p* < 0.001 (***) compared with the control.

MPT pore-opening is triggered by high levels of Ca^2+^ and other stimuli, including oxidants [26]. Proteins such as cyclophilin D, VDAC, and ANT are believed to be involved in the organization of MPT pores [27]. The inhibition of cyclophilin D, pharmacologically or genetically, reportedly attenuates MPT pore formation. The process of mitochondrial permeability transition (MPT) can result in mitochondrial swelling and subsequently lead to cell death, either by apoptosis or necrosis, depending on the specific physiological conditions. Cyclosporin A (CsA) interacts with cyclophilin D, thereby inhibiting the MPT pores [28]. To further elucidate the relationship between mitochondrial Ca^2+^ and MPT, we pretreated A549 cells with CsA before exposing them to 40 µM DP. Notably, CsA exhibited a protective effect against DP-induced cell death by restoring viability and reducing necrotic death (Figure 7A,B). We also observed that 1 µM CsA pretreatment of A549 cells almost completely blocked DP-induced mitochondrial vacuolation (Figure 7C). We utilized the Calcein-AM/CoCl_2_ method to evaluate the induction of MPT by DP in A549 cells. We observed that DP decreased Calcein-AM fluorescence by 40% compared with the control at 12 h. CsA mostly prevented this event (Figure 7D). Sustained MPT pore-opening leads to the loss of MMP resulting in cell death [29]. Confirming the correlation between MMP and the MPT pores, CsA significantly restored DP-induced MMP loss (Figure 7E). Taken together, our results suggest that DP induces cell death by reducing MPT pore activity, leading to the loss of MMP.

### 2.8. DP Induces Ferroptosis in A549 Cells

**Figure 8:**
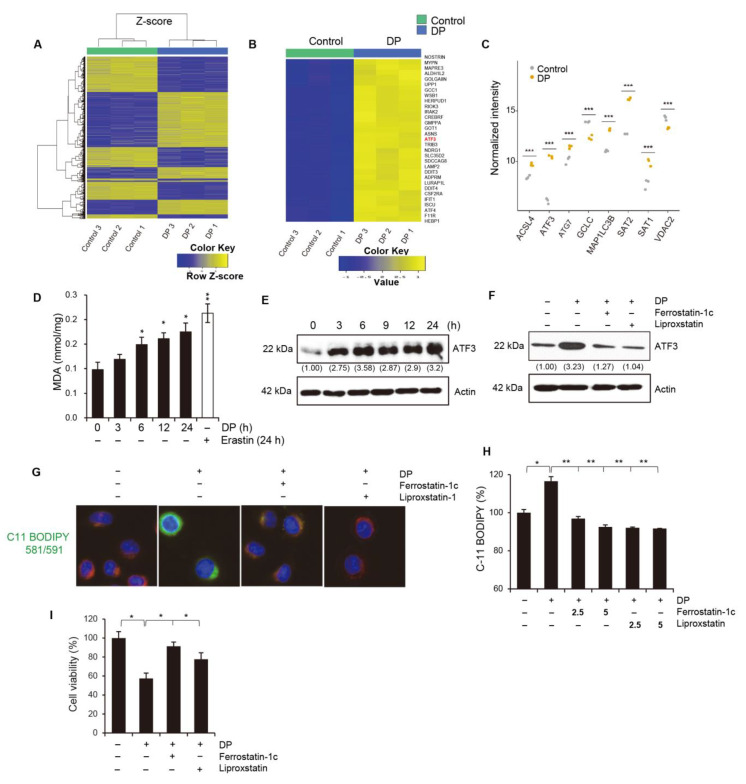
ATF3 is a candidate major gene in DP-induced ferroptosis. (**A**–**C**) Microarray data from A549 cells treated with DP for three independent experiments. (**D**) A549 cells were treated with 40 μM DP for the indicated time, and malondialdehyde (MDA) was measured. A ferroptosis inducer, 10 μM erastin, was used as a positive control. (**E**) A549 cells were treated with 40 μM DP for the indicated time and western blotting was performed to assess ATF3 expression. (**F**) Cells were treated with 40 μM DP for 24 h with 1 h pretreatment with 5 μM ferrostatin-1 and 5 μM liproxstatin-1. Western blotting was performed to assess ATF3 expression. Actin was used as a loading control. Numbers indicate the expression levels of each protein relative to actin. (**G**,**H**) A549 cells were treated with 40 μM DP for 24 h with 1 h pretreatment with 5 μM ferrostatin-1 and 5 μM liproxstatin-1. Cells were assessed for lipid peroxidation using the lipophilic redox-sensitive dye C11-BODIPY 581/591, which shifts fluorescence from red to green in response to oxidation. BODIPY fluorescence intensity was determined by using a fluorescence microscope (**G**) and flow cytometry (**H**). (**I**) A549 Cells were treated with 40 μM DP for 24 h with 1 h pretreatment with 5 μM ferrostatin-1 and 5 μM liproxstatin-1. Cell viability was measured by the MTT assay. All data are expressed as the mean ± SD obtained from at least three independent experiments. Statistical differences were analyzed by two-way ANOVA with Tukey’s multiple comparisons test and presented as *p* < 0.05 (*), *p* < 0.01 (**) and *p* < 0.001 (***) compared with the control.

To investigate the potential genes involved in the formation of vacuoles and MPT-mediated cell death induced by DP, we used a microarray to detect changes in mRNA expression in A549 cells treated with DP (Figure 8A,B). Surprisingly, DP induced the expression of a number of genes related to the regulation of ferroptosis. We reviewed the literature on these candidate genes and selected seven key genes related to ferroptosis (Figure 8C). Among them, ATF3 was the most significantly differentially expressed. To investigate the relationship between MPT-mediated cell death induced by DP and ferroptosis, we examined the involvement of a member of the ATF/CREB family of transcription factors, activation transcription factor 3 (ATF3), which is rapidly induced by various cellular stresses, such as DNA damage, oxidative stress, and cell injury. Ferroptosis is a form of regulated nonapoptotic cell death involving overwhelming iron-dependent lipid peroxidation. Here, we measured whether DP treatment of A549 cells resulted in an increase in lipid peroxides. It was observed that malondialdehyde (MDA), one of the final products of lipid peroxidation in cells, was significantly increased after 6 h of treatment with DP (Figure 8D). We analyzed the expression of ATF3 using Western blotting for each DP treatment time and found that ATF3 was increased in a time-dependent manner by DP (Figure 8E; See Figure S6A for original blots). We further tested whether the ferroptosis inhibitors could block the expression levels of ATF3 and GPX4 (Glutathione peroxidase 4), which is an enzyme and main regulator of ferroptosis. Ferrostatin-1 and liproxstatin-1 restored DP-induced ATF3 expression (Figure 8F; See Figure S6B for original blots). Through C11-BODIPY staining, it was determined whether DP induced lipid peroxidation, and it was confirmed that DP treatment induced lipid peroxidation that was attenuated by ferroptosis inhibitors (ferrostatin-1 and liproxstatin-1) (Figure 8G,H). Finally, we confirmed that the ferroptosis inhibitors could prevent DP-induced cell death in A549 cells (Figure 8I). Taken together, our results suggest that DP induces ATF3-related ferroptosis in A549 cells.

### 2.9. Ferroptosis Inhibition Prevents Mitochondrial Ca^2+^ and MPT Opening

**Figure 9:**
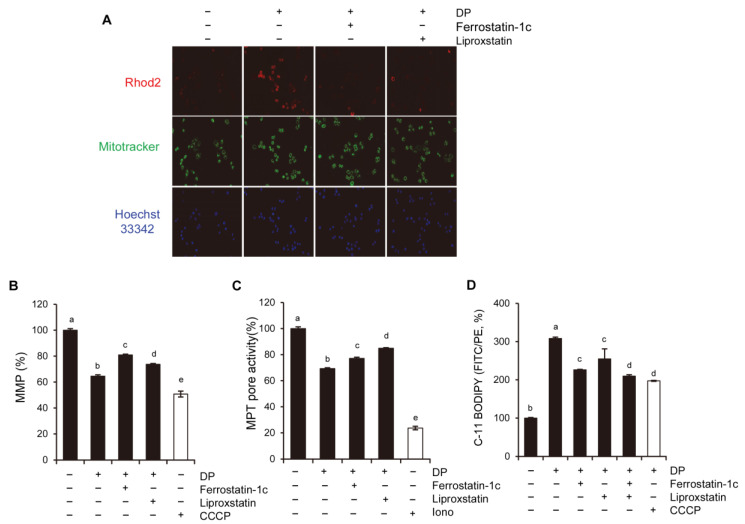
Ferroptosis is involved in DP-induced MPT-mediated cell death. (**A**) A549 cells were treated with 40 μM DP for 24 h with 1 h pretreatment with 5 μM ferrostatin-1 and 5 μM liproxstatin-1. The mitochondrial Ca^2+^ level was detected by Rhod-2 (1 µg/mL) and MitoTracker green (200 nM). Images were acquired using a fluorescence microscope (magnification, 100×, scale bar, 0.1 mm). (**B**) The cells were stained with 50 nM DiOC6(3) for 20 min at 37 °C and then analyzed via flow cytometry. Carbonyl cyanide 3-chlorophenylhydrazone (CCCP; 50 µM) was used as a positive control. (**C**) The cells were pretreated with ferroptosis inhibitors and further treated with 40 μM DP for 6 h, after which MPT was determined using Calcein-AM/CoCl_2_. (**D**) The A549 cells were pretreated with ferroptosis inhibitors, and their C-11 BODIPY fluorescence intensity was assessed using microscopy and flow cytometry. All data are expressed as the mean ± SD obtained from at least three independent experiments. Statistical differences were analyzed by two-way ANOVA with Tukey’s multiple comparisons test. Different lowercase letters above the bar graph indicate significant statistical differences (*p* < 0.05).

To confirm the relationship between the DP-induced ferroptosis and MPT-mediated cell death, the mitochondrial Ca^2+^ levels were measured via Rhod-2 staining. As shown in Figure 9A, the ferroptosis inhibitor almost inhibited mitochondrial Ca^2+^ in DP-induced A549 cells. Indeed, pretreatment with ferroptosis inhibitors significantly restored MMP and MPT pore activity-induced DP (Figure 9B,C). Additionally, we observed that the DP-mediated lipid peroxidation was dramatically suppressed under the ferroptosis inhibitor treatment conditions (Figure 9D). These observations suggest that the DP treatment in the A549 cells induced ferroptosis and caused MPT-derived cell death due to the excessive Ca^2+^ and ROS levels in the mitochondria.

### 2.10. DP Induces Release of HMGB1 That Can Enhance NK Cell Function

**Figure 10:**
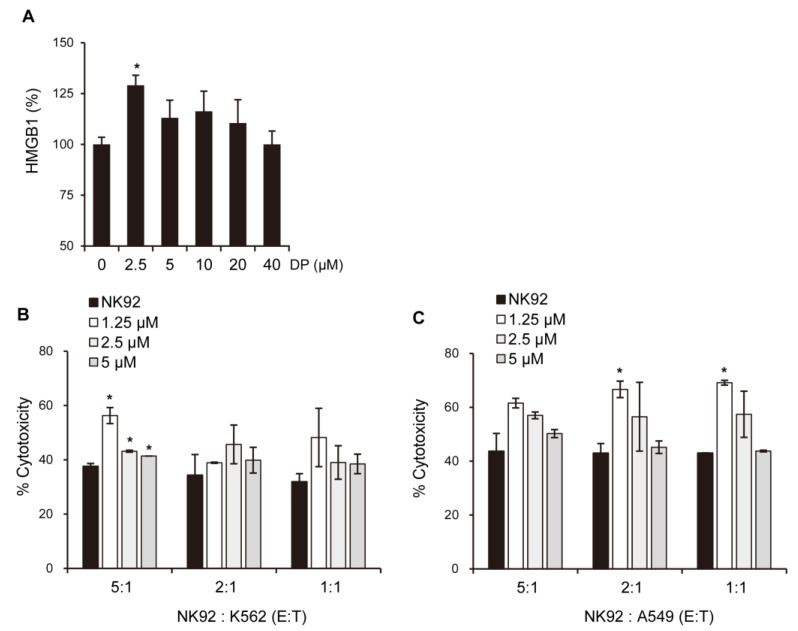
DP releases HMGB1 and enhances cytotoxicity in NK-92 cells. (**A**) The secreted HMGB1/HMG-1 levels were measured using ELISA. (**B**,**C**) To assess the effect of HMGB1-containing supernatant from DP-treated A549 cells on NK cell activity, NK-92 cells were co-cultured with K562 cells and A549 cells. All data are expressed as the mean ± SD obtained from at least three independent experiments. Statistical differences were analyzed by two-way ANOVA with Tukey’s multiple comparisons test and presented as *p* < 0.05 (*) compared with the control.

Recent studies suggest that the secretion of high-mobility group box 1 protein (HMGB1), one of the products of apoptosis by ferroptosis, contributes to the activation of immune cells surrounding cancer cells. DP increased the secretion of HMGB1 in A549 cells (Figure 10A). It is accepted that ferroptosis, as a type of necrotic death, is more immunogenic than apoptosis to induce the release of inflammatory mediators and DAMPs, such as HMGB1, thus rendering the cellular environment a highly proinflammatory state. We conducted an experiment to determine whether exposure to DP increased the secretion of HMGB1 in A549 or K562 cells and thereby increased the cancer cell-killing activity of NK cells. The supernatant of DP-treated A549 or K562 cells was added to the co-culture of NK-92 and A549 or K562 cells, and the increase in the cancer cell-killing activity of NK cells was evaluated. In our experiment, we observed that HMGB1-containing supernatant obtained from DP-treated A549 cells increased the cytotoxicity of NK92 cells in co-culture with K562 cells or A549 cells. These findings are of interest, as they suggest that HMGB1 released by cells treated with DP may have a functional impact on NK cell activity (Figure 10B,C). These findings suggest that HMGB1 could have potential as an immunomodulatory agent for enhancing the anti-tumor activity of NK cells.

## 3. Discussion

DP is a natural compound that is found in the fruit of the *P. tomentosa* tree, also known as the empress tree. It is a type of flavanone, a class of flavonoids that are commonly found in plants and have been associated with various health benefits. DP has been studied for its potential anti-cancer properties, as it has been shown to inhibit the growth and proliferation of various types of cancer cells. It is believed to exert its anti-cancer effects by inhibiting the activity of the ERBB family of receptors, which are commonly overexpressed in many types of cancer [30]. However, more research is required to comprehend the mechanisms of the action and potential therapeutic applications of DP.

First, we measured cell viability for 19 compounds obtained from the fruit of *P. tomentosa* using A549 cells (Supplementary Figure S3). A strong anticancer effect was observed with increasing numbers of compounds, indicating the presence of geranyl and hydroxyl groups. Hydroxyl groups possess binding affinity with the cellular plasma membrane, resulting in enhanced cell death. Moreover, vacuolation could be observed only in the cases of compounds 13, 14, 16, and 18 (Supplementary Figure S4). To determine the difference in cytotoxicity according to the presence or absence of geranyl groups, we measured cytotoxicity using taxifolin (5,7,3′,4′-flavan-on-ol, also known as dihydroquercetin) treatment, in which the geranyl group was excluded from the DP structure. As a result of the experiment, vacuole formation and cytotoxicity could not be observed under the taxifolin treatment conditions (Supplementary Figure S5).

Programmed non-apoptotic cell death encompasses various mechanisms and phenotypes, including cell death with vacuoles (autophagy, entosis, methuosis, and paraptosis), cell death dependent on mitochondria (mitoptosis and parthanatos), iron-dependent cell death (ferroptosis), cell death with immune reactions (pyroptosis and NETosis), and other types, such as necroptosis [31]. In this study, we aimed to investigate the involvement of MPT-mediated cell death and ferroptosis in the formation of vacuoles in A549 lung epithelial cells. MPT-mediated cell death is characterized by rapid increases in inner mitochondrial membrane permeability, and it involves the cyclophilin D-dependent opening of MPT pores. Although MPT-mediated cell death has been described in the context of neuronal excitotoxicity and ischemia-reperfusion injury (IRI), it has not been extensively studied. MPT-mediated cell death is characterized by cell swelling, the induction of an inflammatory response, increased intracellular Ca^2+^ levels, massive ATP depletion, and increased ROS levels. Ca^2+^ is known to contribute to mitochondrial permeability disruption, which leads to reduced ATP production and increased ROS levels, and could also lead to the further perturbation of mitochondrial function and lysosomal permeability. Physiological mitochondrial Ca^2+^ is attributed to the activation of either the MPT pore or MCU. The regulation of the mitochondrial Ca^2+^ levels is primarily dependent on the mitochondrial calcium uniporter (MCU) located on the inner mitochondrial membrane, while the cyclophilin D located in the mitochondrial matrix plays a crucial role in the MPT opening that leads to mitochondrial-dependent apoptosis or necrosis. Furthermore, ROS produced by the mitochondria and subsequent oxidative damage are strongly associated with MPT, and the selective inhibition of cyclophilin D using cyclosporine A can rescue cell death by preventing MPT. We speculate that the MCU may play a role in MPT regulation, and this regulatory relationship may be significant in MPT-mediated cell death. Although our results demonstrated that DP promoted MPT-mediated cell death, we believe that another regulated cell death mechanism could be a trigger of DP-induced cell growth inhibition, as DP can enhance immune responses.

Ferroptosis, a newly discovered form of regulated cell death, is caused by the accumulation of lipid-based ROS. Current studies have shown that ferroptosis is associated with multiple pathological processes, including ischemia-reperfusion injury in the liver, heart, and kidney, as well as neurodegenerative diseases, such as Alzheimer’s, Parkinson’s, and Huntington’s diseases [32]. Other diseases, such as chronic obstructive pulmonary disease (COPD), acute lung injury, and liver fibrosis, are also linked to ferroptosis. The main morphological features of ferroptosis include increased mitochondrial membrane densities and ruptured outer mitochondrial membranes, while the nucleus remains unaffected. Ferroptosis is also characterized by iron accumulation, lipid peroxidation, and increased MMP. Recent studies have identified Frataxin, glutathione peroxidase 4 (GPX4), HSPB1, SLC7A1, and NCOA4 as key regulators of ferroptosis by modulating iron homeostasis and mitochondrial functioning [33]. Specifically, System xc^−^ is responsible for transporting extracellular cystine into the cell, where it is converted into cysteine for the synthesis of glutathione (GSH). GSH peroxidase 4 (GPX4) is an enzyme that can directly catalyze the reaction between GSH and lipid hydroperoxides, reducing the cellular level of lipid peroxidation. However, the depletion of GSH or inhibition of GPX4 can lead to the accumulation of lipid hydroperoxides [34]. In this study, we used ferroptosis inhibitors to investigate the relationship between DP and ferroptosis. We observed that treatment with ferroptosis inhibitors increased the MPT pore activity levels and significantly restored MMP induced by DP. Taken together, our results suggest that DP induces not only MPT-mediated cell death, but also ferroptosis, and that both of these mechanisms are influenced by each other.

ATF3, activating transcription factor 3, has been shown to play a role in ferroptosis by regulating the expression of genes involved in iron metabolism and lipid peroxidation. ATF3 is activated in response to cellular stress, such as endoplasmic reticulum (ER) stress, amino acid deprivation, and oxidative stress. It has been shown to be involved in the regulation of several cellular processes, including the unfolded protein response (UPR), amino acid metabolism, and autophagy. In ferroptosis, ATF3 has been shown to upregulate the expression of several genes involved in iron metabolism, such as transferrin receptor 1 (TfR1) and divalent metal transporter 1 (DMT1), which are responsible for iron uptake. ATF3 also upregulates the expression of genes involved in lipid peroxidation, such as ACSL4, which is responsible for the esterification of polyunsaturated fatty acids (PUFAs) in phospholipids and subsequent lipid peroxidation. Our study demonstrated that the upregulation of ATF3 is significantly involved in DP-induced ferroptosis. In future studies, it is necessary to investigate the specific subgenes regulated by ATF3 and their respective functions.

The immunological response depends on molecules presented or released by dying cells, and the HMGB1 protein, which is a nuclear DNA-binding factor and a secreted protein, plays a crucial role. Chemotherapeutics and cellular stress can promote the translocation of HMGB1 from the nucleus to the cytosol and subsequent release into the extracellular space. In the present study, DP was found to increase the secretion of HMGB1 in A549 cells (Figure 9A). Interestingly, DP-induced supernatant with A549 cells enhanced the cytotoxic activity of NK-92 cells (Figure 10B,C). Recent studies suggest that ferroptosis, through lipid peroxidation-mediated plasma membrane rupture, can lead to sterile inflammation by generating damage-associated molecular patterns (DAMPs) that stimulate pattern recognition receptor (PRR)-expressing immune cells, resulting in an inflammatory process called necroinflammation, if regulated necrosis initiated immune activation. While the precise mechanisms of this process are still unclear, Fer-1, a small-molecule inhibitor, has been shown to reduce immune cell infiltration in models of acute kidney injury and decrease cytokine and chemokine expression levels (such as C-X-C-motif chemokine 2, interleukin 6, p65 subunit of NF-κB, interleukin 33, TNF-α, and monocyte chemotactic protein 1), suggesting that ferroptotic DAMPs can induce secondary immune cell activation and cytokine production. Nevertheless, further research is required to determine which DP-induced factors in the A549 cells stimulated the cytotoxic ability of the NK-92 cells. Taken together, our findings suggest that DP induces MPT-derived cell death through ferroptosis and can increase the cytotoxic effect of NK-92 cells, providing a novel mechanism for ferroptosis involving MPT-mediated cell death. In summary, our findings reveal that DP treatment hinders cell proliferation by initiating ferroptosis and MPT-related cell death in A549 cells. We aim to provide valuable foundational research data for addressing non-small-cell lung cancer resistant to apoptosis through this study.

## 4. Materials and Methods

### 4.1. Reagents and Material

We purchased propidium iodide (PI), C11-BODIPY, MitoTracker, and 3,3′-Dihexyloxacarbocyanine iodide (DiOC6-3) from Thermo Fisher Scientific (Waltham, MA, USA), while chloroquine (CQ), N-acetyl-L-cysteine (NAC), 3-(4,5-dimethyl-2-thiazolyl)-2,5-diphenyl-2H-tetrazolium bromide (MTT), carbonyl cyanide m-chlorophenylhydrazone (CCCP), ferrostatin-1c, liproxstatin, ionomycin, and MitoTEMPO were obtained from Sigma-Aldrich (Saint Louis, MO, USA). The caspase-8, caspase-9, and caspase-3 colorimetric assay kits and z-VAD-fmk (a pan-caspase inhibitor) were purchased from R&D systems (Minneapolis, MN, USA). We purchased Wortmannin and U0126 from Tocris (Bristol, UK), and 2′7′-dichlorofluorescein diacetate (H_2_DCF-DA), BAPTA-AM, CsA, and Ru360 were obtained from Calbiochem (San Diego, CA, USA). Additionally, we purchased dihydroethidium (Hydroethidine, HE), Fluo4-AM, MitoSOX, and BAPTA from Invitrogen (Carlsbad, CA, USA), Rhod-2 and Ruthenium Red from Abcam (Cambridge, UK), and glutathione (GSH) from Duchefa Biochemie (Haarlem, The Netherlands). The MitoProbe Transition Pore Assay Kit was purchased from Molecular Probes (Eugene, OR, USA), and the Human HMGB1/HMG-1 ELISA kit was obtained from Novus Biologicals (Centennial, CO, USA). Calcein-AM (ultra-pure) was purchased from Enzo Life Sciences (Farmingdale, NY, USA). The FITC-conjugated TUNEL Assay Kit (ab66108) and lipid peroxidation (MDA) assay kit (ab118972) were acquired from Abcam.

### 4.2. Plant Material

Sample collection for this study was as follows: the same *Paulownia tomentosa* mature fruit as sample KRIB 0059121-0059123 collected in Sancheng, Republic of Korea, in June 2015 was recollected in June 2021 by Doo-Young Kim (Natural Product Central Bank, Korea Research Institute of Bioscience and Biotechnology).

### 4.3. Extraction and Isolation

The target compound was isolated from dried mature fruit of *P. tomentosa* as previously described [16]. To obtain DP, the plant material containing the mature fruit was dried and ground into a fine powder. The compound was then extracted from the material using 100% CH_3_OH, concentrated in vacuo at 40 °C, and purified using methods such as column chromatography (silica gel, reversed-phase silica gel, and prep-HPLC). Brief, the CHCl_3_ layer (30.0 g) was fractionated on a silica gel column (10 × 30 cm, 230–400 mesh, 700 g) and eluted using hexane−EtOAc mixtures (15:1 (1.5 L)→1:1 (1 L), and pure EtOAc (2 L)) to produce 20 pooled fractions (Fr. 1–20), which were combined based on a comparison of their LC profiles. Subfraction Fr. 18 (0.8 g) enriched with DP was separated with a Kinetex Biphenyl column using a gradient of CH_3_OH−H_2_O (71%→100%) to produce DP (89.2 mg). The purity (>98%) of each isolated DP was confirmed by UPLC-PDA and MS.

### 4.4. Cell Culture

The human lung cancer A549 and human leukemia cell lines were obtained from the American Type Culture Collection (ATCC). The cells were cultured in DMEM medium (Welgene, Korea) supplemented with 10% fetal bovine serum (Gibco, Rockville, MD, USA) and 1% penicillin–streptomycin (Gibco, Rockville, MD, USA) at 37 °C under 5% CO_2_ atmosphere and with 100% humidity. The human NK-92 cell line was cultured in α-MEM (without ribo- and deoxyribonucleosides) supplemented with 2 mM L-glutamine, 1.5 g/L sodium bicarbonate, 0.2 mM inositol, 0.02 mM folic acid, 0.1 mM 2-mercaptoethanol (Sigma, St. Louis, MO, USA), 12.5% FBS (Gibco, Rockville, MD, USA), 12.5% horse serum (Gibco, Rockville, MD, USA), and 200 U/mL IL-2 (PeproTech, Rocky Hill, NJ, USA). NK-92-sensitive target cell line K562 is a human chronic myelogenous leukemia (CML) cell line. The K562 cell line was cultured RPMI-1640 medium supplemented with 10% fetal bovine serum and 1% penicillin/streptomycin.

### 4.5. Cell Viability

A549 cells were seeded at a density of 1 × 10^5^ cells/mL in 24-well plates and incubated for 24 h before being treated with various concentrations of DP for designated periods of time. The cells were incubated with an MTT solution (final concentration of 0.5 mg/mL) for 30 min after treatment. Next, the resulting purple formazan was dissolved in DMSO, and the absorbance was measured at 540 nm using an ELISA plate reader (Epoch BioTek, Winooski, VT, USA).

### 4.6. Caspase Activity

Commercially available total colorimetric caspase-3, caspase-8, and caspase-9 assay kits were used to assess their activity in A549 cells treated with different concentrations of DP. A549 cells were first cultured overnight, treated with varying concentrations of DP, lysed, and equal amounts of protein were incubated with each substrate (DEVE-PNA, IETD-AFC, or LEHD-PNA). The reaction mixtures were then analyzed on a microplate reader with absorbance measured at 405 nm. Each sample was tested in duplicate according to the manufacturer’s instructions.

### 4.7. TUNEL Assay

Following a 24 h treatment with 40 μM DP, A549 cells were fixed onto glass slides to retain their structure and keep the DNA fragments intact. The cells were then exposed to a permeabilizing solution, Triton X-100. The TdT enzyme was added to the A549 cells along with the FITC-labeled dUTPs. The TdT enzyme catalyzes the incorporation of the labeled dUTPs into the 3’-OH ends of the fragmented DNA. Cells containing fluorescein-labeled dUTPs were directly examined under a fluorescence microscope.

### 4.8. Measurement of Cytosolic and Mitochondrial Ca^2+^ Levels

Once harvested, A549 cells were exposed to either Fluo-4 AM or Rhod-2 AM at a concentration of 1 µg/mL, followed by incubation at 37 °C for 15 min, washing with HBSS, and immediate analysis using the FITC or PE channels on a flow cytometer (CytoFLEX, Beckman Coulter, Brea, CA, USA).

### 4.9. Transmission Electron Microscopy

A total of 1 × 10^5^ A549 cells/mL were cultured in 6-well plates for 1 and 6 h and subsequently treated with 30 µM DP. The cells were then collected and fixed with a primary solution containing 2.5% paraformaldehyde and 2.5% glutaraldehyde. The sections were then stained with uranyl acetate and were visualized by Cryo-TEM (JEM-1400 Plus, 120 kV) (Jeol, Tokyo, Japan).

### 4.10. Intracellular ROS Level Measurement

The levels of ROS were measured using H_2_DCF-DA, HE, and MitoSOX (mitochondrial superoxide indicator) probes. Approximately 5 × 10^4^ cells/mL were seeded onto a 24-well plate and treated with DP. Following treatment, the cells were collected by trypsinization, suspended in 0.6 mL of PBS, and incubated with 2 μM He, 4 μM H_2_DCF-DA, or 1 μM MitoSOX for 20 min in the dark at 37 °C. The fluorescence was measured using a flow cytometer (CytoFLEX, Beckman Coulter, CA, USA) on the FL-2 channel for HE and the FL-1 channel for H_2_DCF-DA and MitoSOX.

### 4.11. PI Flow Cytometric Detection

After the indicated treatment, A549 cells were incubated with PI dye (1 mg/mL) in the dark at 37 °C for 20 min. The cells were then washed with PBS and analyzed using the PE channel on a flow cytometer (CytoFLEX, Beckman Coulter, CA, USA).

### 4.12. Hoechst 33342, Rhod-2, and MitoTracker Stainings

A549 cells were seeded in 24-well plates on coverslips. Next, the cells were treated with DP for 24 h. After treatment, the cells were washed with PBS and incubated with pre-warmed Hoechst for 20 min at 37 °C in the dark. The cells were then washed with PBS again and visualized under a fluorescence microscope (ZEISS, Jena, Germany).

### 4.13. Live Cell Imaging

A549 cells were seeded in an 8µ-chamber and stained with Rhod-2 and MitoTracker green to measure the mitochondrial calcium levels. The images were taken using a fluorescence microscope (ZEISS, Germany) at ×60 magnification.

### 4.14. Lipid Peroxidation Measurement

To measure lipid oxidation, we used the C11-BODIPY 581/591 probe. A549 cells were seeded in a 12-well microplate at a density of 1 × 10^5^ cells/well and incubated with C11-BODIPY 581/591 (1 µM) in RPMI-1640 for 30 min. The fluorescence signals of green (484/510 nm) and red (581/610 nm) C11-BODIPY were recorded using CytoFLEX flow cytometry (Beckman Coulter, Brea, CA, USA). MDA assays were performed according to the supplier’s instructions. RFU was measured at Ex/Em = 532/553 nm using a microplate reader.

### 4.15. Mitochondrial Membrane Potential Assay

After the indicated treatment, A549 cells were incubated with DiOC6(3) dye (40 nM) for 20 min at 37 °C in the dark. The cells were then washed with PBS and observed under a flow cytometer (CytoFLEX, Beckman Coulter, CA, USA) using the FITC channel. To serve as a positive control, CCCP, a mitochondrial oxidative phosphorylation uncoupler, was used.

### 4.16. Western Blotting

The cells were washed with ice-cold PBS and subsequently lysed on ice for 30 min in lysis buffer (Pro-Prep, iNtRON) containing a protease inhibitor. After the collected supernatants were quantified using the Bradford method following centrifugation at 13,200 rpm for 25 min at 4 °C, the lysates were boiled for 5 min at 95 °C and separated using SDS-PAGE gels. The separated proteins were then transferred onto polyvinylidene difluoride membranes (PVDF, Millipore, MA, USA), which were blocked with 3% skim milk for 30 min to prevent nonspecific binding before incubating with specific primary and secondary antibodies. After washing the membrane three times with TBS-T for 30 min, immune complex signals were detected using enhanced chemiluminescence (ECL, Thermo, USA), and equal protein amounts were assessed using anti-actin as an internal control.

### 4.17. MPT Pore Activity Measurement

The MitoProbe transition pore assay kit (Molecular Probes, Eugene, OR, USA) was used to determine MPT by utilizing calcein acetoxymethyl ester (AM) and CoCl_2_. The technique involves calcein-AM, which is a nonfluorescent molecule that accumulates in the cytosol and mitochondria, and is cleaved by intracellular esterases to produce the fluorescent calcein dye that does not cross the plasma or mitochondrial membranes. The addition of CoCl_2_ quenches the fluorescence of cytosolic, but not mitochondrial calcein in healthy cells. However, in cells with MPT, CoCl_2_ enters the mitochondria to quench the mitochondrial calcein fluorescence. The calcein-AM/CoCl_2_ method is restricted to vital cells as it requires the activity of intracellular esterases. For this assay, the cells (1 × 10^5^) were labeled with 10 nM calcein-AM, 400 μM CoCl_2_, and 0.5 μM ionomycin for 15 min at 37 °C. After washing the cells with HBSS/Ca, they were resuspended in HBSS/Ca and analyzed using CytoFLEX flow cytometry. Calcein-AM fluorescence was expressed as a percentage of its initial value by the following calculation: (mean calcein fluorescence of cells treated with Calcein-AM and CoCl_2_/mean calcein fluorescence of cells treated with Calcein-AM) × 100. The positive control used for this assay was ionomycin, which is known to induce complete MPT.

### 4.18. HMGB1 ELISA Assay

The experimental procedures were carried out following the guidelines provided in the assay kit’s manual. Briefly, A549 cells were treated with various concentrations of DP (ranging from 0 to 40 μM) and the resulting supernatant was collected. Next, the supernatant was added to the wells of the microplate, followed by the addition of HRP-conjugated anti-HMGB1 antibody and overnight incubation. The wells were then aspirated and washed several times with a wash buffer, after which TMB substrate was added and incubated. Stop solution was then added, and the contents of the wells were mixed by gentle shaking. Finally, the optical density was measured at 450 nm using a microplate reader immediately after completing the assay.

### 4.19. Measurement of NK Cell Cytotoxicity

To assess the effect of HMGB1-containing supernatant from DP-treated A549 cells on NK cell activity, A549 cells were treated with 1.25, 2.5, and 5 μM DP for 24 h, and the resulting supernatant was collected. The supernatant was then incubated with NK-92 cells for 4 h before co-culturing with K562 cells and A549 cells at E:T ratios of 5:1, 2:1, and 1:1. After 24 h of co-culturing, cytotoxicity was evaluated using Calcein-AM staining. The fluorescence intensity emitted from the lysed target cells was measured by a fluorescence plate reader.

### 4.20. Statistical Analysis

All data are presented as the mean ± standard deviation of three independent experiments unless otherwise described. The differences were determined by two-way ANOVA with Tukey’s multiple comparison test using GraphPad Prism (version 6). The statistical significance compared with the control group or between the two groups is indicated as follows: *p* < 0.05 (*), *p* < 0.01 (**), and *p* < 0.001 (***). In case there were three or more comparison groups, they were written in lowercase letters. Different lowercase letters above the bar graphs indicate significant statistical differences (*p* < 0.05).

## Data Availability

All data analyzed in this study are available from the corresponding author (mokim@kribb.re.kr) on reasonable request.

## Supplementary Materials

The following supporting information can be downloaded at: https://www.mdpi.com/1422-0067/24/8/7057/s1.
