# Supplementary material for: Diplacone Isolated from Paulownia tomentosa Mature Fruit Induces Ferroptosis-Mediated Cell Death through Mitochondrial Ca2+ Influx and Mitochondrial Permeability Transition
Source: Int J Mol Sci. 2023 Apr 11;24(8):7057. doi: 10.3390/ijms24087057 (PMC10138418; doi:10.3390/ijms24087057)
Supplement: Supplementary file 1 [file ijms-24-07057-s001.zip › ijms-2314887-supplementary.pdf]

Supplementary Information

Diplacone Isolated from *Paulownia tomentosa* Mature Fruit Induces Ferroptosis-Mediated Cell Death through Mitochondrial Ca^{2+} Influx and Mitochondrial Permeability Transition

Eun Sol Oh *et al.*,

● Supplementary Figures

Figure S1-1. Fractionation and analysis from mature fruits of *Paulownia tomentosa* 4

Figure S1-2. ^{1}H and ^{13}C NMR spectrum of diplacone 5

Figure S1-3. UV, MS/MS, MS and HREIMS data of diplacone 6

Figure S1-4. HPLC-DAD spectrum of isolated diplacone (Purity ≥98%).. 6

Figure S2. Diplacone-induced cell growth inhibition is not associated with autophagy 7

Figure S3. Effect of 19 compounds obtained from the fruit of *P. tomentosa* on A549 cells............... 8

Figure S4. Compounds that can induce vacuoles.. 9

Figure S5. Effect of Taxifolin on cell viability and vacuolation 10

Figure S6. Original western blots in Figure 8 11

Experimental Section

The melting point was obtained using a Stuart SMP40 automatic melting point apparatus (Bibby Scientific, Stone, Staffordshire, UK). Optical rotations were measured on a JASCO P-1020 digital polarimeter (JASCO, Easton, MD, USA) with a 100 mm glass microcell. UV spectra were recorded on a SpectraMax M5 Multi-Mode Microplate Reader (Molecular Devices, Sunnyrale, CA, USA). ECD spectra were recorded on a Jasco J-815 CD spectrometer. IR spectra were obtained on a Tensor 27 FT-IR spectrometer (Bruker, Billerica, MA, USA) with KBr pellets. 1D (^{1}H, ^{13}C, and DEPT) and 2D (COSY, HMQC, and HMBC) NMR spectra were obtained on Bruker AM 400 and Bruker DRX 500 spectrometers (Bruker) with tetramethylsilane (TMS) as the internal standard. HRESIMS were measured on an ultraperformance liquid chromatography quadrupole time of flight mass spectrometer (UPLC-QTOF-MS, Waters, Milford, MA, USA) in the negative-ion mode. Medium-pressure liquid chromatography (MPLC) was performed with a Spot Prep II 250 instrument (Armen, Paris, France). Preparative HPLC was performed using a PLC 2020 personal purification system (Gilson, Middleton, WI, USA). Column chromatography (CC) was performed using a silica gel (40-63 μm, Silicyle, Québec, Canada) and a Cosmosil 75 C_{18} Prep resin (75 μm, Nacalai Tesque, Kyoto, Japan). Preparative chromatography was performed using a YMC Actus Triart C_{18} column (5 μm, 250 × 20 mm, YMC, Kyoto, Japan), YMC Pack Pro C_8 column (5 μm, 250 × 20 mm, YMC), YMC Pack ODS AQ HG column (10 μm, 250 × 20 mm, YMC), Inno C_{18} column (5 μm, 250 × 20 mm, Young Jin Biochrom, Seongnam, Korea), Kinetex Biphenyl column (5 μm, 250 × 21.2 mm, Phenomenex, Torrance, CA, USA), or a Synergi polar RP column (4 μm, 250 × 21.2 mm, Phenomenex).

UPLC-QTof-MS analysis

P. tomentosa extract profiling was performed using an ACQUITY UPLCTM system (Waters Corporation, Milford, MA, USA) equipped with a binary solvent delivery manager and a sample manager coupled to a Micromass Q-TOF Premier^{TM} mass spectrometer (Waters Corporation) with electrospray ionization (ESI) interface with MassLynex V4.1 software. Chromatographic separation

was performed using an ACQUITY BEH C18 chromatography column (2.1 × 100 mm, 1.7 μm). The column temperature was maintained at 35°C, and the mobile phases A and B were water with 0.1% formic acid and acetonitrile with 0.1% formic acid, respectively. The gradient elution program was as follows: 0.0-1.0 min, 5% B; 1.0-10.0 min, 5-30% B; 10.0-10.5 min, 30-98% B; wash for 1.5 min with 98% B; and a 3.0 min recycle time. The injection volume was 2.0 μL, and the flow rate was 0.4 mL/min. The mass spectrometer was operated in positive ion mode. N_2 was used as the desolvation gas. The desolvation temperature was 350°C, the flow rate was 500 L/h, and the source temperature was 100°C. The capillary and cone voltages were 2300 V and 35 V, respectively. The Q-TOF Premier^{TM} was operated in v mode with a 9000 mass resolving power. The data were collected for each test sample from 100 to 1500 Da with 0.25-s scan time and 0.01-s interscan delay over the 15 min analysis time. Leucine-enkephalin was used as the reference compound (*m/z* 554.2661 in the negative mode).

Diplacone (16)

colorless powder; mp > 124℃; ^{1}H NMR (500 MHz, CD_3OD) δ 1.45 (3H, s, H-10″), 1.51 (3H, s, H-9″), 1.64 (3H, s, H-4″), 1.84 (2H, m, H-5″), 1.94 (2H, m, H-6″), 2.57 (1H, dd, J = 2.8, 17.1 Hz, H-3b), 2.94 (1H, dd, J = 12.8, 17.1 Hz, H-3a), 3.11 (2H, d, J = 7.1 Hz, H-1″), 4.95 (1H, t, H-7″), 5.09 (1H, t, H-2″), 5.13 (1H, dd, J = 2.7, 12.8 Hz, H-2), 5.83 (1H, s, H-8), 6.68 (2H, s, H-5′, 6′), 6.81 (1H, s, H-2′). ^{13}C NMR (125 MHz, CD_3OD) δ 16.6 (C-4"), 18.1 (C-10"), 22.2 (C-1"), 26.3 (C-9"), 28.2 (C-6"), 41.3 (C-5"), 44.7 (C-3), 80.9 (C-2), 95.9 (C-8), 103.6 (C-4a), 110.1 (C-6), 115.1 (C-2'), 116.7 (C-5'), 119.7 (C-6'), 124.4 (C-2"), 125.9 (C-7"), 132.4 (C-1'), 132.4 (C-8"), 135.7 (C-3"), 146.9 (C-3'), 147.3 (C-4'), 162.8 (C-8a), 162.9 (C-5), 166.4 (C-7), 198.2 (C=O, C-4); HREIMS, *m/z* 423.1844 (calcd for $C_{25}H_{28}O_6$ 424.1886).

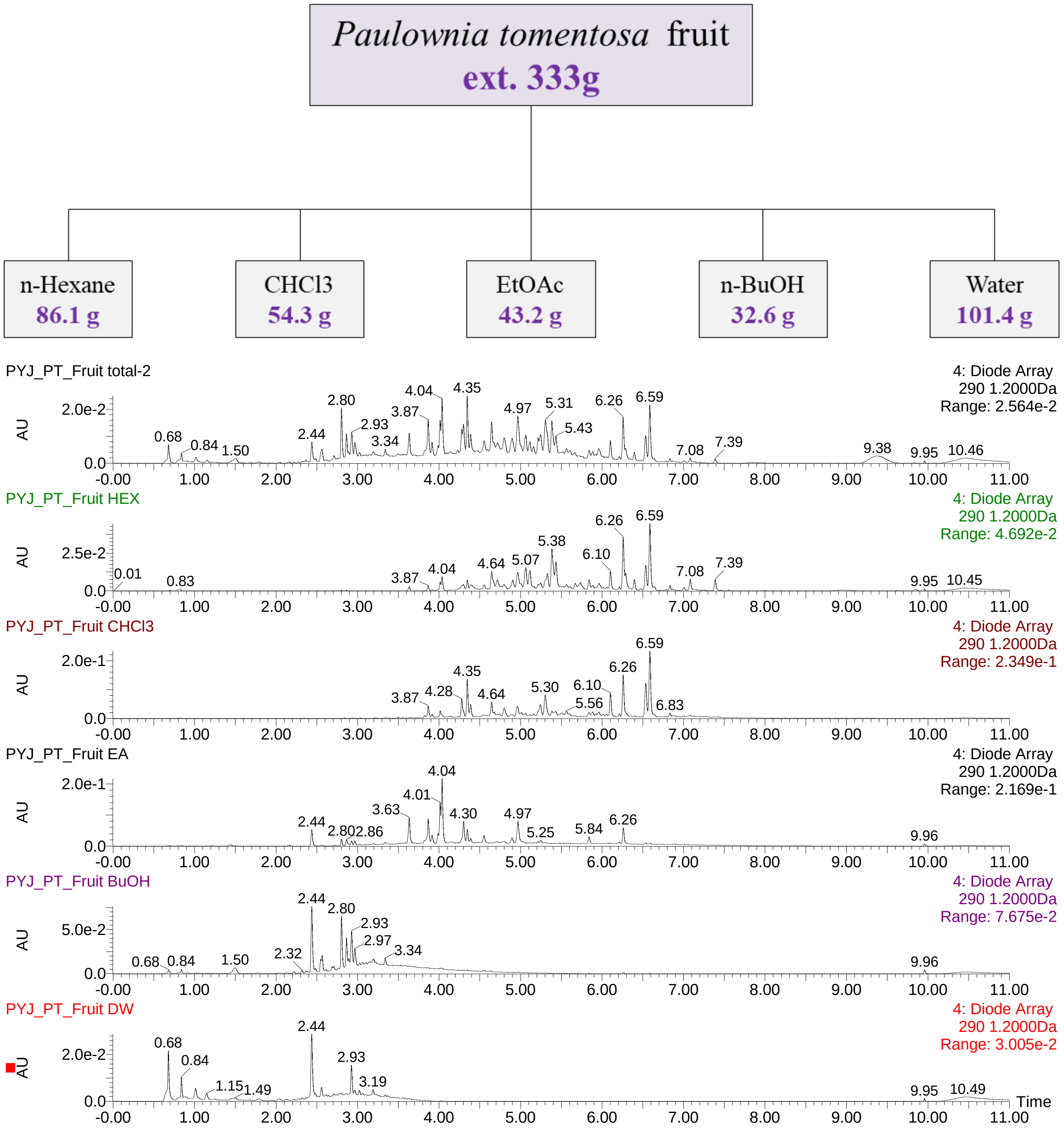

Figure S1-1. Fractionation and UPLC-PDA analysis from mature fruits of *Paulownia tomentosa*.

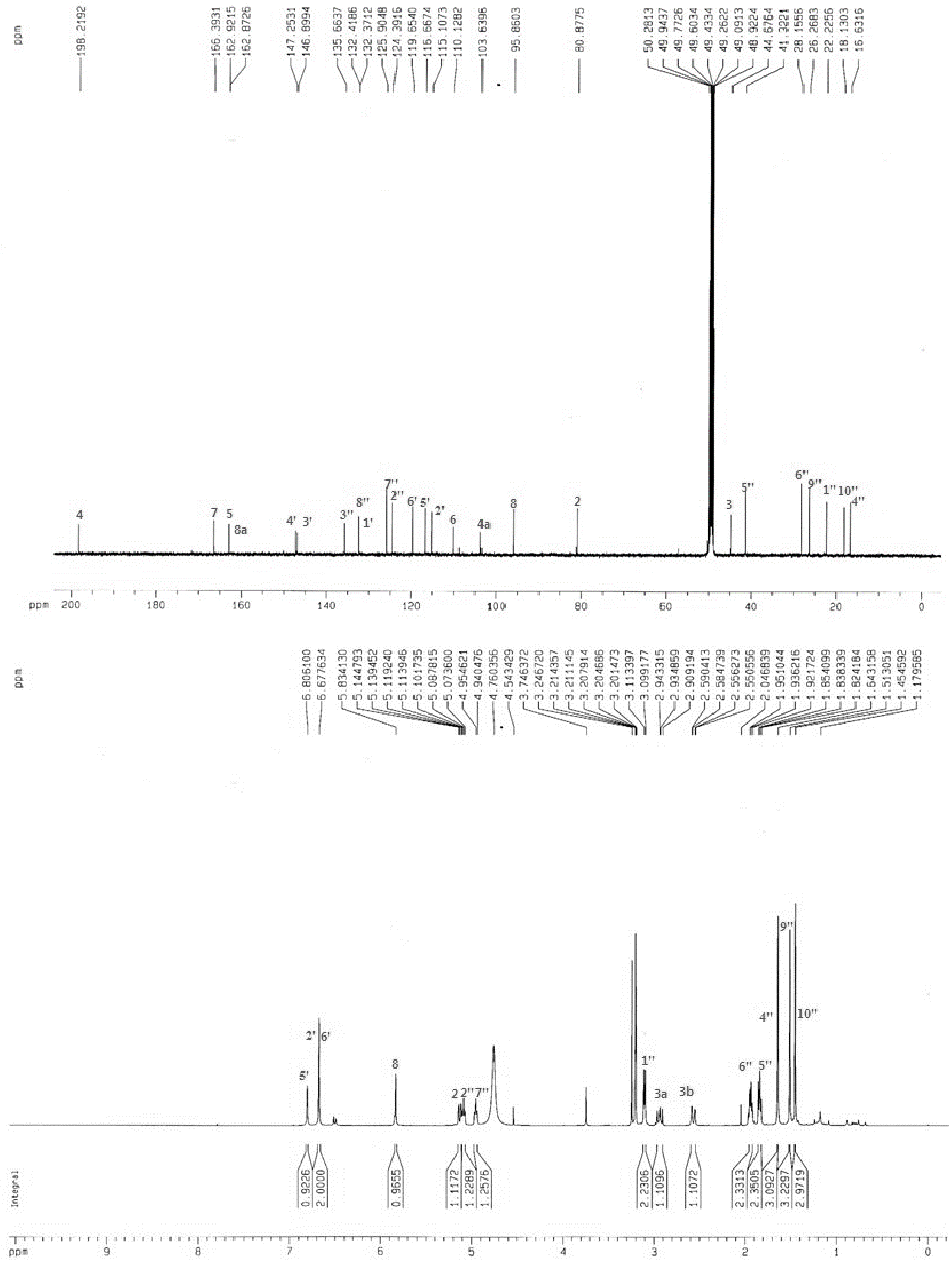

Figure S1-2. ^{1}H and ^{13}C NMR spectrum of diplacone.

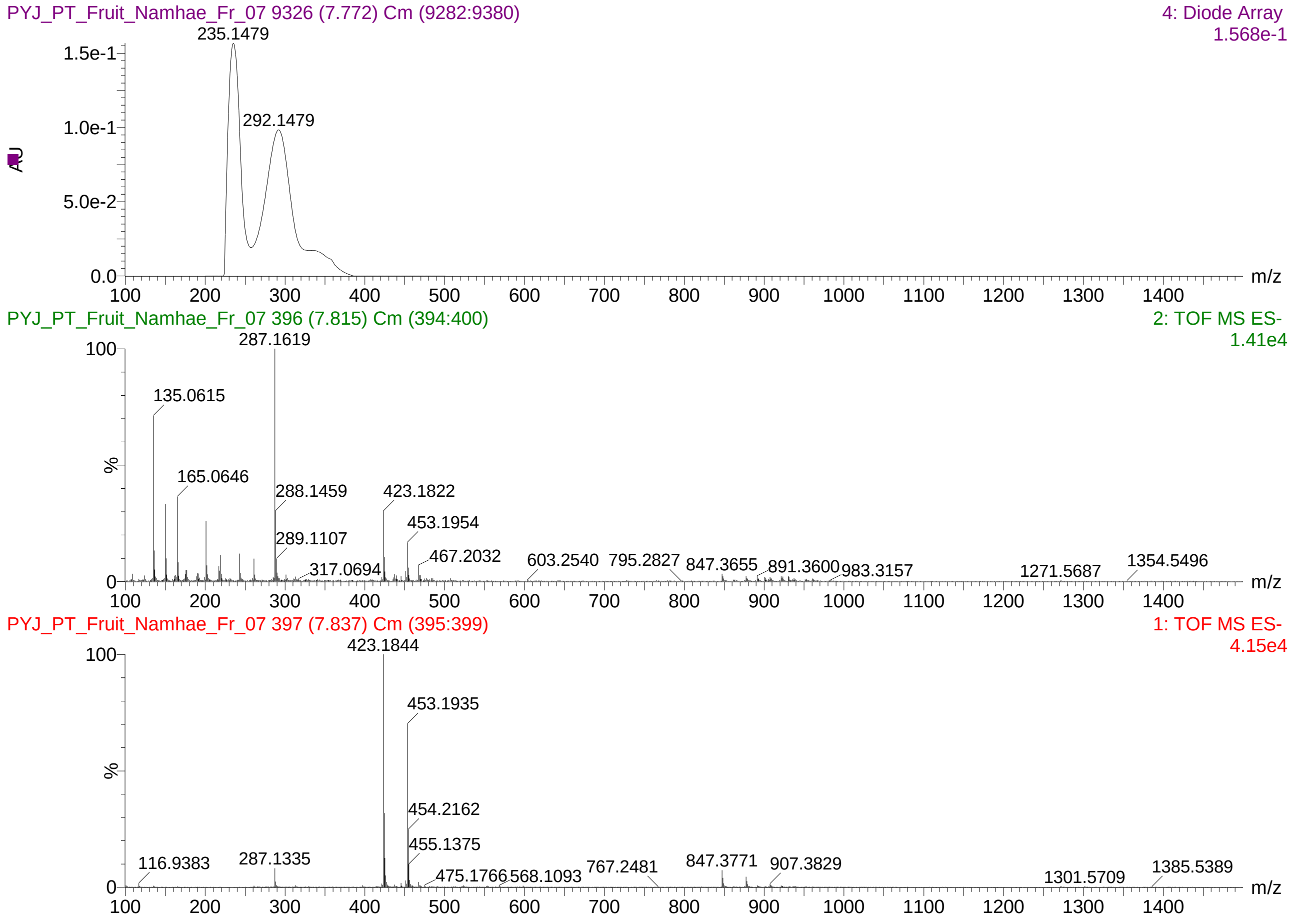

Figure S1-3. UV, MS/MS, and MS data of diplacone.

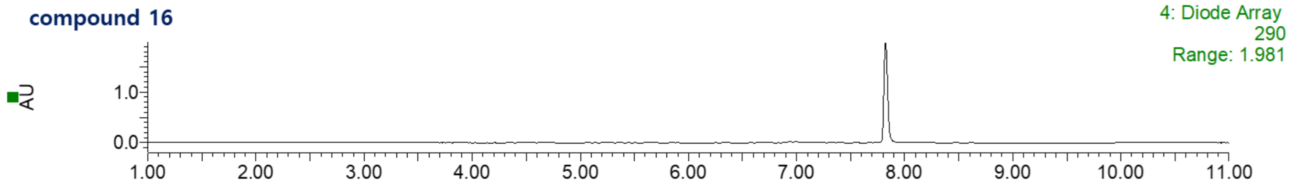

Figure S1-4. HPLC-DAD spectrum of isolated diplacone (Purity ≥98%).

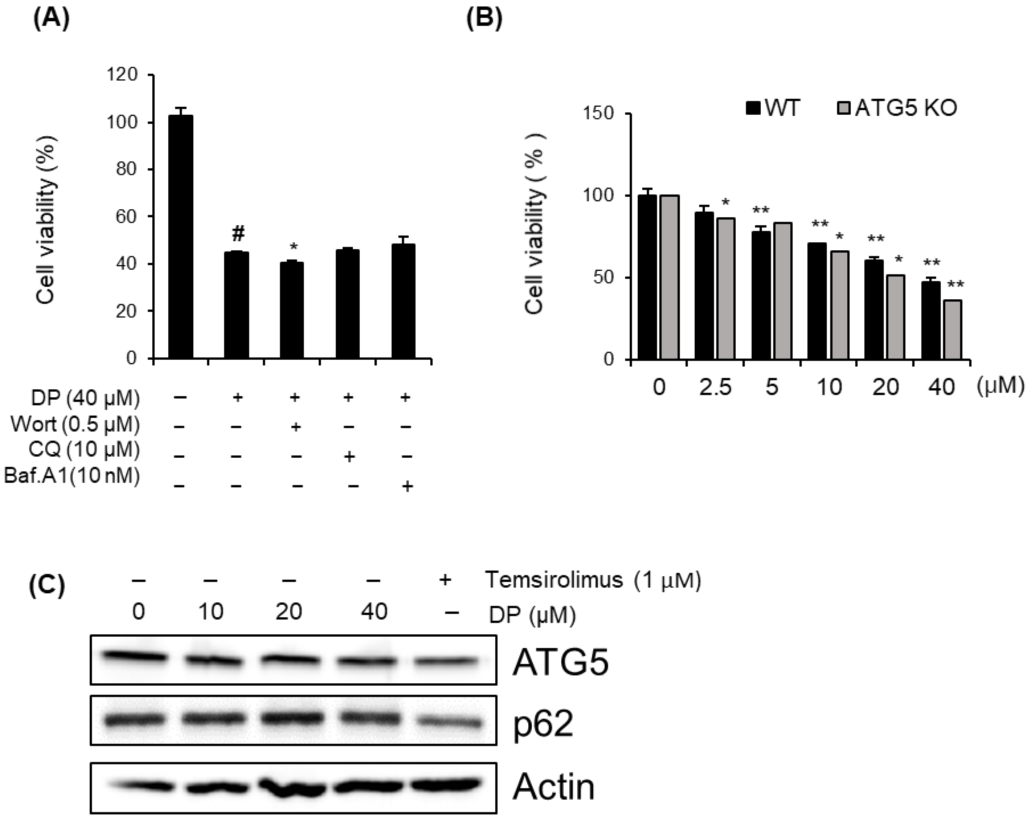

Figure S2. DP-induced cell growth inhibition is not associated with autophagy. (A) Autophagy inhibitors Wortmannin, chloroquine (CQ), and bafilomycin A1 (Baf.A1) were pretreated for 1 hour, and 40 μM DP was added for 24 hours. Cell growth was analyzed using the MTT assay. (B) ATG5 knock out cell line was treated with DP and then cell growth was analyzed using the MTT assay. (C) A549 cells were treated with indicated concentration of DP for 24 hours, and western blotting was performed to assess ATG5 and p62 expression. Actin was used as a loading control. Error bars indicated the average ± standard deviation of three independent experiments. #, $p < 0.01$ (compared with the vehicle control group); *, $p < 0.05$ and **, $p < 0.01$ (compared with the group with DP treat only).

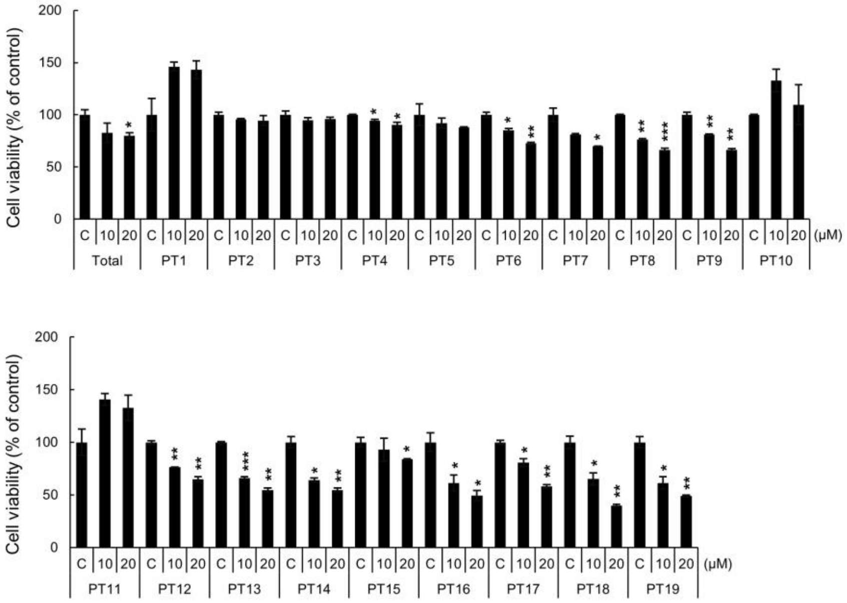

Figure S3. Effect of 19 compounds obtained from the fruit of *P. tomentosa* on A549 cells. A549 cells were treated with 19 compounds obtained from the fruit of *P. tomentosa* for 24 hours, and cell viability was analyzed by MTT. Bar graphs indicated the average ± standard deviation of three independent experiments. *, $p < 0.05$, **, $p < 0.01$ and ***, $p < 0.001$ (compared with control).

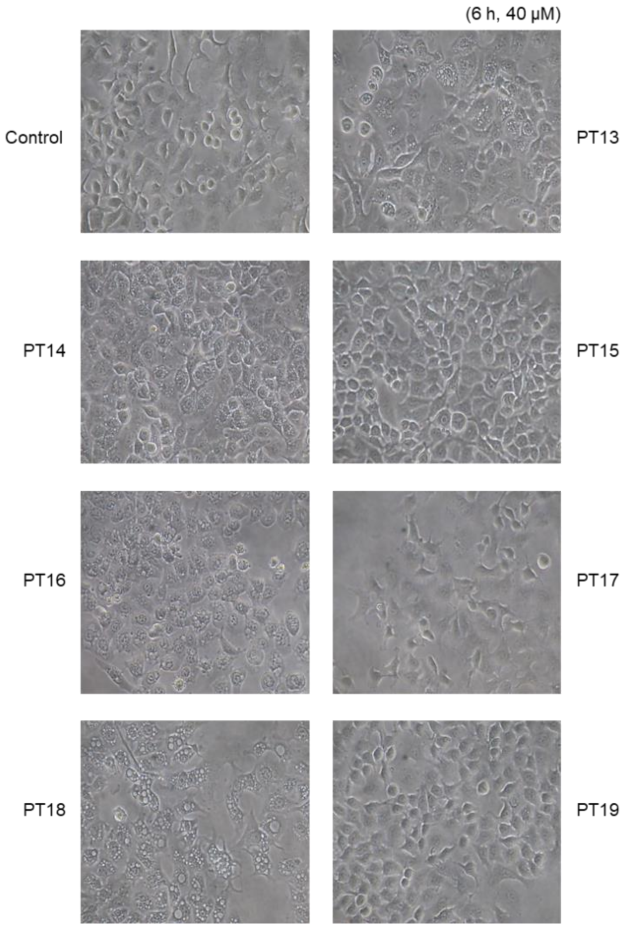

Figure S4. Compounds that can induce vacuoles. Compounds 13, 14, 16, and 18 obtained from the fruit of *P. tomentosa* were treated with 40 μM for 24 hours, and intracellular vacuole formation was observed under an optical microscope (magnification, ×200).

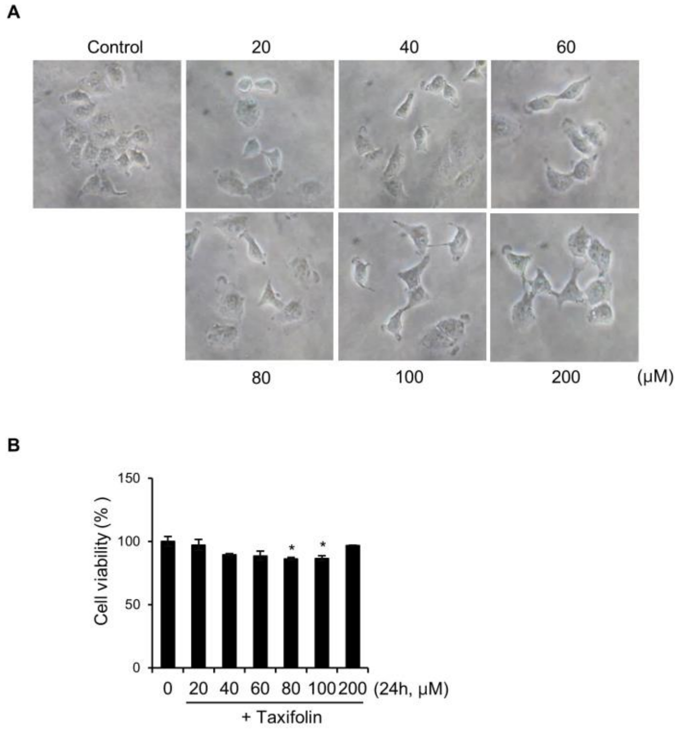

Figure S5. Effect of Taxifolin on cell viability and vacuolation. Taxifolin (5,7,3′,4′-flavan-on-ol, also known as dihydroquercetin) was obtained by removing the geranyl group from the DP compound. Taxifolin was treated by concentration, and cell shape (A) and cell growth rate (B) were analyzed. Bar graphs indicated the average ± standard deviation of three independent experiments. *, $p < 0.05$ (compared with control).

A

Target	Size	Original image
ATF3	21 kDa	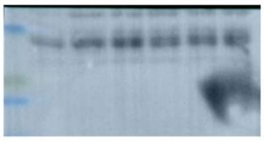
Actin	42 kDa	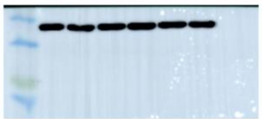

B

Target	Size	Original image
ATF3	21 kDa	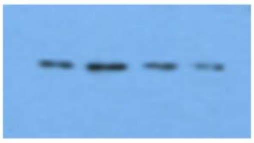
Actin	42 kDa	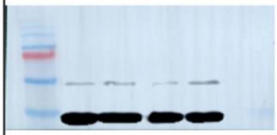

Figure S6. Original western blots in Figure 8. **(A)** Uncropped and unprocessed images of Figure 8E. **(B)** Original images of Figure 8F.
